# A Rad53 Independent Function of Rad9 Becomes Crucial for Genome Maintenance in the Absence of the RecQ Helicase Sgs1

**DOI:** 10.1371/journal.pone.0081015

**Published:** 2013-11-20

**Authors:** Ida Nielsen, Iben Bach Bentsen, Anni H. Andersen, Susan M. Gasser, Lotte Bjergbaek

**Affiliations:** 1 Department of Molecular Biology and Genetics, Aarhus University, Aarhus, Denmark; 2 Friedrich Miescher Institute for Biomedical Research, Basel, Switzerland; Universita' di Milano, Italy

## Abstract

The conserved family of RecQ DNA helicases consists of caretaker tumour suppressors, that defend genome integrity by acting on several pathways of DNA repair that maintain genome stability. In budding yeast, Sgs1 is the sole RecQ helicase and it has been implicated in checkpoint responses, replisome stability and dissolution of double Holliday junctions during homologous recombination. In this study we investigate a possible genetic interaction between *SGS1* and *RAD9* in the cellular response to methyl methane sulphonate (MMS) induced damage and compare this with the genetic interaction between *SGS1* and *RAD24*. The Rad9 protein, an adaptor for effector kinase activation, plays well-characterized roles in the DNA damage checkpoint response, whereas Rad24 is characterized as a sensor protein also in the DNA damage checkpoint response. Here we unveil novel insights into the cellular response to MMS-induced damage. Specifically, we show a strong synergistic functionality between *SGS1* and *RAD9* for recovery from MMS induced damage and for suppression of gross chromosomal rearrangements, which is not the case for *SGS1* and *RAD24*. Intriguingly, it is a Rad53 independent function of Rad9, which becomes crucial for genome maintenance in the absence of Sgs1. Despite this, our dissection of the MMS checkpoint response reveals parallel, but unequal pathways for Rad53 activation and highlights significant differences between MMS- and hydroxyurea (HU)-induced checkpoint responses with relation to the requirement of the Sgs1 interacting partner Topoisomerase III (Top3). Thus, whereas earlier studies have documented a Top3-independent role of Sgs1 for an HU-induced checkpoint response, we show here that upon MMS treatment, Sgs1 and Top3 together define a minor but parallel pathway to that of Rad9.

## Introduction

The maintenance of genome stability is a fundamental aspect of all life. DNA is damaged regularly by both exogenous factors and endogenous events. In order to maintain a stable genome, cells have devoted a significant percentage of their genome to encode DNA repair, checkpoint and other DNA damage tolerance proteins that act in multiple complexes and interrelated pathways to protect the genome.

One such group of enzymes is the conserved family of RecQ DNA helicases, which are genome caretakers. They exert multiple cellular functions in order to suppress genome instability, and loss of RecQ helicase activity is associated with a hyperrecombination phenotype. In human cells, five members of the RecQ helicase family have been identified. Mutations in three of the genes are responsible for genetic disorders (Blooms, Werneŕs and Rothmund-Thomson syndromes) that correlate with enhanced frequencies of chromosomal rearrangements and aneuploidy. At a clinical level this is manifested as a cancer predisposition, or, in the case of Werneŕs syndrome, premature aging (reviewed in [1], [2]).

Sgs1 is the RecQ helicase in *S. cerevisiae*, encoded by the non-essential gene *SGS1*. Loss of Sgs1 leads to increased rates of chromosome missegregation and mitotic hyper-recombination [3], [4]. Furthermore, *sgs1Δ* mutants confer sensitivity to DNA damaging agents such as MMS and the deoxynucleotide depleting agent HU [5], [6].

RecQ helicases functions in a complex with Rmi1 and DNA topoisomerase III (Top3). Top3 enzymes are type 1A DNA topoisomerases, which have the unique ability to unlink single-stranded DNA catenanes that may arise during homologous recombination or at sites of termination of DNA replication [7]. Loss of this function probably accounts for the severe slow growth or lethality of *top3*-deficient cells in budding yeast, fission yeast or mouse [3], [8], [9]. The slow growth of the budding yeast *top3Δ* mutant is accompanied by hyper-recombination and chromosome loss [10]. Surprisingly enough, the most severe consequences of *TOP3* deletion in yeast can be suppressed by deletion of *SGS1*, although cells retain the abnormally high recombination rates that accompany *sgs1Δ* mutation [3]. Knowing that Sgs1, the fission yeast RecQ helicase Rqh1, and the human homologue BLM interact physically with DNA topoisomerase III, the suppression may be explained if RecQ helicase activity promotes an accumulation of improperly resolved exchange events in the absence of Top3, thereby engendering genomic instability.

Consistent with these roles, it was shown that the absence of either Sgs1 or Top3 is synthetic lethal with the loss of other enzymes or proteins implicated in DNA repair, recombination and/or replication [11], [12], [13]. Because many of these negative interactions can be suppressed by eliminating homologous recombination altogether, it has been proposed that the Sgs1/Top3 complex functions downstream of homologous recombination [11], [14]. Indeed both BLM and Sgs1 were shown to function together with Top3 to resolve crossover structures induced by recombination to suppress reciprocal exchange [15], [16].

Sgs1 also functions upstream of break-induced strand exchange and is replication fork-associated both under normal conditions and when replication forks stall due to HU [17]. Indeed, Sgs1 acts to maintain DNA polymerases stably associated with stalled replication forks on HU [17], [18], and is particularly important in the presence of a mutant ATR kinase allele, called *mec1*-100 [19]. Furthermore, it was shown that Sgs1 and Top3 act in the same pathway for polymerase stability during HU treatment [18]. One explanation for this would be that the combined action of Sgs1/Top3 reverses pathological pairing events at stalled forks. In addition to this we have reported a Top3-independent role for Sgs1 in the checkpoint response to HU [18], which may entail the recruitment of Rad53 [20]. Indeed, the contribution of Sgs1 to Rad53 activation is independent of its helicase activity and mechanistically distinct from its role in DNA polymerase stabilisation [20]. In budding and fission yeast, the intra-S phase checkpoint in response to MMS is partially compromised by deletion of *SGS1* or *Rqh1+* [5], [21]. It was further reported that deletion of yeast *TOP3* also impairs Rad53 activation in response to MMS [22]. This observation contrasts with results on HU, where no significant role for Top3 in Rad53 activation was found [18].

As RecQ helicases are multifunctional enzymes it is not a surprise that many interaction partners have been identified for these enzymes. Among the proteins BLM is found in complex with is BRCA1, another prominent caretaker protein [23]. BRCA1 binds directly to branched DNA structures and four-way junctions, a feature shared with the RecQ helicase family, and it is thought to control recombination events in the cell (reviewed in [24]). There is no obvious *BRCA1* homologue in *S. cerevisiae*, however, the *RAD9* gene does share homology and several features with *BRCA1*. Both proteins contain BRCT domains, which are heavily engaged in protein-protein interactions, and they both get hyperphosphorylated in a checkpoint dependent manner. Rad9 was the first checkpoint protein identified and it is thought to act at an early step of damage recognition to activate the central checkpoint kinases Rad53 and Chk1 [25], [26], [27], [28]. Rad9 works as an adaptor in the damage checkpoint response, as it is phosphorylated in a Mec1/Tel1-dependent manner. This damage checkpoint also relies on the PCNA-like clamp complex Ddc1-Rad17-Mec1 (in higher eukaryotes the 9-1-1 complex for Rad9, Rad1 and Ddc1), which is loaded near DNA damage by the Rad24-clamp. The phosphorylated form of Rad9 specifically interacts with Rad53, enhancing its activation by Mec1. This suggests that Rad9 recruits Rad53 and Chk1 to DNA damage-dependent complexes [26], [27].

Rad9 is required for maintaining genome stability particularly in the case of strand breakage, like BRCA1. Absence of Rad9 leads to increased rates of spontaneous chromosome loss and rearrangements [29], [30], [31], [32]. This could be readout for loss of proper checkpoint function, however accumulating evidence suggests that Rad9 may play multiple roles in the DNA damage response. For instance, Rad9 has been implicated in nucleotide excision repair of UV-damaged DNA [33], [34], for suppression of mutagenic post-replicative repair during MMS induced damage [35], and a post checkpoint activation role for Rad9 in promoting efficient repair of DSBs imposed by irradiation has also been suggested [36]. Thus, it seems we are far from understanding the complexity of the cellular functions that Rad9 may be engaged in to act as a caretaker gene.

In this study we examine the genetic interaction between *RAD9* and *SGS1* in response to MMS-induced lesions. We find that recovery after MMS exposure for the *sgs1Δrad9Δ* double mutant is more compromised than for the *sgs1Δrad24Δ* double mutant, strongly suggestive of a Rad24 independent pathway of Rad9 for genome maintenance in the absence of Sgs1. This is furthermore reflected in MMS-induced genomic instability monitored as gross chromosomal rearrangements (GCR), where the *sgs1Δrad9Δ* mutant has a significant higher rate than *sgs1Δrad24Δ*. We show that this cannot be explained by a more severe checkpoint defect for the *sgs1Δrad9Δ* mutant as Rad53 and Chk1 activation is equally compromised in *sgs1Δrad9Δ* and *sgs1Δrad24Δ* backgrounds. Using a Rad9 mutant defective in Rad53 activation, we are able to show that in the absence of Sgs1 recovery and suppression of GCR after MMS exposure requires a Rad53 independent function of Rad9. This strongly indicates a role for Rad9 in genome maintenance, which is checkpoint independent and crucial in the absence of Sgs1.

## Materials and Methods

### Yeast strains and general yeast manipulation

All strains used are listed in Table 1 and are derived from either S288C for GCR assays or W303 for all other assays. The W303 strains carry a *rad5* point mutation that weakens its activity. Unless stated otherwise, the genomic deletions of *SGS1* and *RAD51* were done using a *sgs1*-3::*TRP1* plasmid [37] and a *rad51::URA3* plasmid [38], which both create null alleles. All other deletions are complete ORF deletions based on pFA6a PCR disruption cassette [39], [40]. Double and triple mutants were in many cases obtained by genetic crossing. Growth was at 30°C in YPD unless otherwise indicated. The rad9^7xA^ strains were generated by digesting plasmid Ylp-Rad9^7xA^-HA (kindly provided by Dr. David F. Stern) with SnaBI, which was then used for transformations in the respective strains.

**Table 1:** *Saccharomyces cerevisiae* strains used in this study.

Strain	Genotype	Source
LBy-1	MAT**a**, *ade2*-1, *trp1*-1, *his3*-11, -15, *ura3*-1, *leu2*-3, -112, *can1*-100	R. Rothstein (W303-1A)
LBy-2	MAT**α**, *ade2*-1, *trp1*-1, *his3*-11, -15, *ura3*-1, *leu2*-3, -112, *can1*-100	R. Rothstein (W303-1B)
LBy-3	MAT**a**, *ade2*-1, *trp1*-1, *his3*-11, -15, *ura3*-1, *leu2*-3, -112, *can1*-100, *pep4*::*LEU2*	R. Rothstein (W303-1A)
LBy-7	LBy-3 with *top3*::*HIS3*	Bjergbaek et al., 2005
LBy-8	LBy-3 with *top3*::*HIS3*, *sgs1*-3::*TRP1*	Bjergbaek et al., 2005
LBy-27	LBy-3 with *top3*::*HIS3, rad9::TRP1*	This study
LBy-28	LBy-3 with *top3*::*HIS3*, *rad24*::*URA3*	Bjergbaek et al., 2005
LBy-36	LBy-3 with *sgs1*-3::*TRP1*, *rad24*::*URA3*	Bjergbaek et al., 2005
LBy-40	LBy-3 with *top3*::*HIS3*, *sgs1*-3::*TRP1*, *rad24*::*URA3*	Bjergbaek et al., 2005
LBy-44	LBy-3 with *sgs1*-3::*TRP1*, *rad9*::*HIS3*	Bjergbaek et al., 2005
LBy-129	LBy-3 with *sgs1*-3::TRP1	Bjergbaek et al., 2005
LBy-316	LBy-1 with *rad9*::*LEU2*	D. Shore (S114)
LBy-366	LBy-1 with *CHK1-13myc-HIS3*	This study
LBy-372	LBy-1 with *CHK-13myc-KanMX sgs1*-3::*TRP1*	This study
LBy-374	LBy-1 with *CHK-13myc-KanMX rad9::TRP1*	This study
LBy-376	LBy-1 with *CHK-13myc-KanMX sgs1*-3::*TRP1 rad9::HIS3*	This study
LBy-383	MAT**a**, *CAN1*, *hxt13*::*URA3*	Lengronne (E1557)
LBy-388	LBy-383 with *sgs1*::*HIS3*	This study
LBy-389	LBy-383 with *rad9*::*TRP1*	This study
LBy-390	LBy-1 with *CHK1-13myc-HIS3 rad24::URA3*	This study
LBy-391	LBy-1 with *rad24*::*URA3*	This study
LBy-400	LBy-383 with *sgs1*::*HIS, rad9*::*TRP1*	This study
LBy-406	LBy-383 with *rad24*::*TRP1*	This study
LBy-407	LBy-383 with *sgs1*::*HIS3, rad24*::*TRP1*	This study
LBy-471	LBy-1 with *rad9-^7xA^-HA-TRP1*	This study
LBy-472	LBy-1 with *rad9-^7xA^-HA-TRP1 sgs1*-3::*TRP1*	This study
LBy-473	LBy-383 with *rad9-^7xA^-HA-TRP1*	This study
LBy-474	LBy-383 with *rad9-^7xA^-HA-TRP1 sgs1::HIS3*	This study
LBy-1085	LBy-1 with *CHK1-13myc-HIS3 rad24::URA3 sgs1::LEU2*	This study

### In situ auto-phosphorylation assay (ISA)

Cultures were grown to 0.5×10^7^ cells/ml at 30°C in YPD prior to α-factor (Lipal Biochem, Zürich, CH) synchronisation during one generation. Cells were sedimented, washed and released into prewarmed YPD medium containing 0.02% MMS. All steps of ISA are as described [41], except 5 µCi/ml γ^32^P-ATP was used. In order to obtain equal loading, protein concentration was determined for each sample by Coomassie blue. Samples were loaded on 10% SDS-polyacrylamide gels, along with 5 µl of a standard (standard, Std), containing a known amount of activated Rad53. Dried filters were exposed for equal times on a Biorad Phosphorimager. After exposure, filters were probed with antibody against RnaseH42 (kindly provided by U. Wintersberger, Vienna, Austria) to provide a quantifiable loading control and allow comparison among different gels and mutants. All experiments were performed 2–3 times with similar results.

### FACS

During the time course of the experiments for Rad53 autophophorylation 1 ml aliquots were taken for FACS. Cells were fixed overnight in 70% ethanol at 4°C. Cells were recovered by centrifugation and incubated 3 hr at 37°C in 0.2 mg/ml RNaseA/50 mM Tris-Cl (pH 7.4). Cells were sedimented and resuspended in 10 µg/ml propidium iodide/50 mM Na-citrate (pH 7.0) and incubated overnight in the dark at 4°C, prior to analysis on a Becton Dickinson FACSCalibur.

### MMS recovery assay

Cultures (0.5×10^7^) were blocked in G1 by α-factor and released into fresh YPD containing different concentrations of MMS (v/v). Cultures were incubated at 30°C for 70 min. Aliquots were then diluted, washed and sonicated prior to plating in triplicate on YPD and incubated at 30°C for 3 days. Survival ratios for a culture without MMS were set as 100%. The recovery assays shown are an average of 2–3 experiments with standard deviations below 10%.

### Gross Chromosomal Rearrangements

GCR analysis was performed as described by Myung and Kolodner [42]. In brief, cells were cultured in YPD to a cell density of 2×10^7^ cells/ml. 10 ml cultures were used for GCR experiments without drug whereas 5 ml cultures were used when MMS was present. Cells were then washed twice with distilled water and suspended in a volume of distilled water equal to the starting culture volume either without or with 0.02% MMS for 2 h at 30°C. This MMS concentration resulted in 10% survival for the strain LBy-400, which displayed the highest MMS sensitivity of the strains tested. After treatment, cells were washed with distilled water twice, suspended in 50 or 100 ml YPD depending on the initial volume, and incubated at 30°C until the culture reached saturation. Different dilutions of the culture were plated on YPD plates in order to determine cell concentration and the rest were plated on plates containing 1 mg/ml 5-FOA and 60 µg/ml of canavanine (FC-plates) to determine GCR frequency. Experiments were conducted with 3–4 individual colonies from each strain, and repeated 2–3 times. Mutation rates were calculated by fluctuation analysis using the method of the median [43]. Spontaneous GCR was measured in the same way just without addition of MMS.

### Chk1 upshift assay

Upshift assays were preformed from TCA extract prepared from cultures arrested in G1 by α-factor treatment and then released into S phase in the presence of 0.1% MMS. Samples for TCA precipitation were taken at the indicated time points. Extract were loaded on 8% SDS-PAGE and Western blotting was performed with monoclonal anti-cMyc antibody (Santa Cruz).

### Immunoprecipitations

To verify the contructed *rad9* ^7xA^ strains immunoprecipitations were performed with anti-HA antibody (Santa Cruz) on yeast extracts obtained from cells grown either in the presence or absence of MMS. Immunoprecipitations were conducted as described in [17]. Samples were analyzed for Rad9 upshift on 8% SDS-PAGE. For the *rad9^7xA^* we load more of our IP material on the SDS-gels to make sure that any slight upshift in the material will be visible.

## Results

### Rad9 is required for recovery after MMS exposure in the absence of the Sgs1/Top3 complex

The relative importance of repair and checkpoint proteins can be measured by determining survival of mutants after increasing doses of DNA damaging agents or replication fork inhibiting agents. Use of survival or recovery assays also enables a dissection of genetic interactions by scoring whether double mutants show epistatic, additive or synergistic effects for survival or recovery.

It is already known that both Sgs1 and Rad9 are required for proper recovery after exposure to MMS. Thus in order to reveal a possible genetic interaction between *SGS1* and *RAD9*, we compared recovery after exposure to MMS between *sgs1Δ*, *rad9Δ* and *sgs1Δrad9Δ* cells. In these assays we also included *RAD24*, as it has previously been reported that *sgs1Δ* and *rad24Δ* show an additive defect for recovery after MMS exposure [5]. Synchronised cultures of single and double mutants were released into S phase in the presence of different concentrations of MMS for 70 min, and recovery was monitored by scoring colony growth on drug-free media.

**Figure 1:**
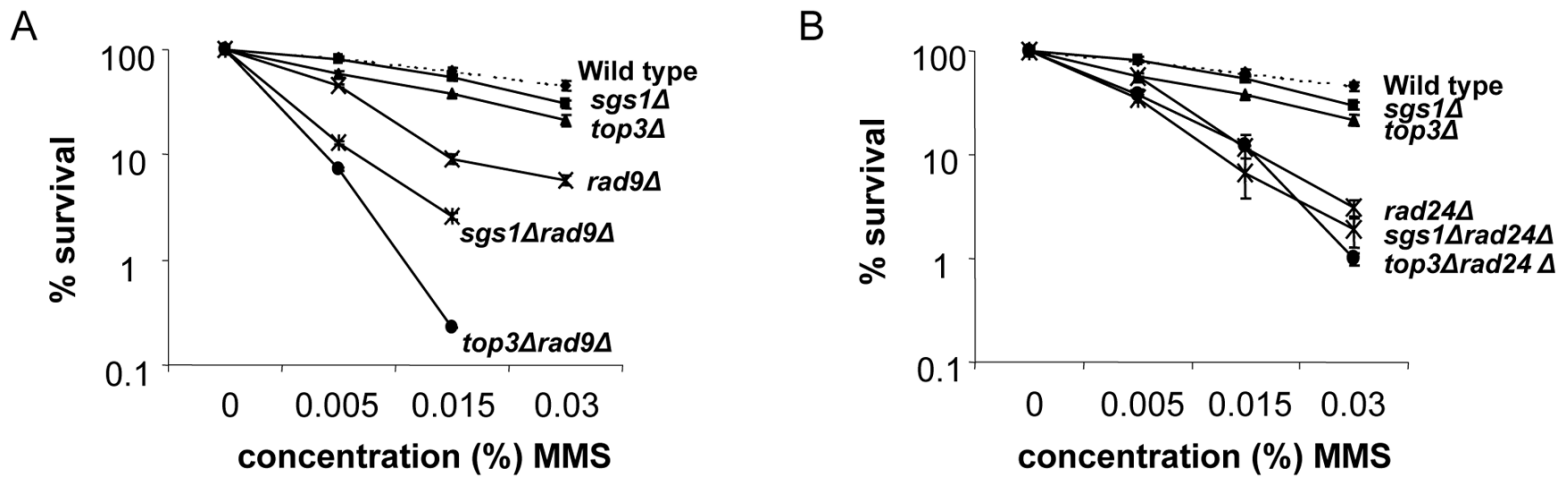
Efficient recovery from MMS reveals a strong synergistic functionality between Sgs1/Top3 and Rad9. (**A**) Survival was monitored as described in Materials and Methods after 70 min exposure to different concentrations of MMS for the indicated strains: wild type (LBy-1), *sgs1Δ* (LBy-129), *top3Δ* (LBy-7), *rad9Δ* (LBy-316), *sgs1Δ rad9Δ* (LBy-44) and *top3Δ rad9Δ* (LBy-27). (**B**) Survival as in A for isogenic strains *rad24Δ* (LBy-391), *sgs1Δ rad24Δ* (LBy-36) and *top3Δ rad24Δ* (LBy-28). The wild type, *sgs1Δ* and *top3Δ* survival curves are added in for comparison from A.

Loss of Rad9 impairs survival but to a lesser extent than a *rad24Δ* deletion (compare Fig. 1A and Fig. 1B). Intriguingly, the *sgs1Δrad9Δ* and *top3Δrad9Δ* double mutants show synergistic defect for recovery, as no survivors are recovered on 0.03% MMS in the double mutants (Fig. 1A). This places Sgs1/Top3 on a survival pathway that is different from that of Rad9. In contrast, the *top3Δrad24Δ* and *sgs1Δrad24Δ* double mutations seem to be additive with respect to loss of survival (fig. 1B).

In conclusion, in the absence of Sgs1/Top3, cells rely heavily on Rad9 for recovery after MMS exposure, and this role of Rad9 is more crucial than the role of Rad24.

### *sgs1Δ* induced genomic instability increases dramatically in *rad9Δ* cells

**Figure 2:**
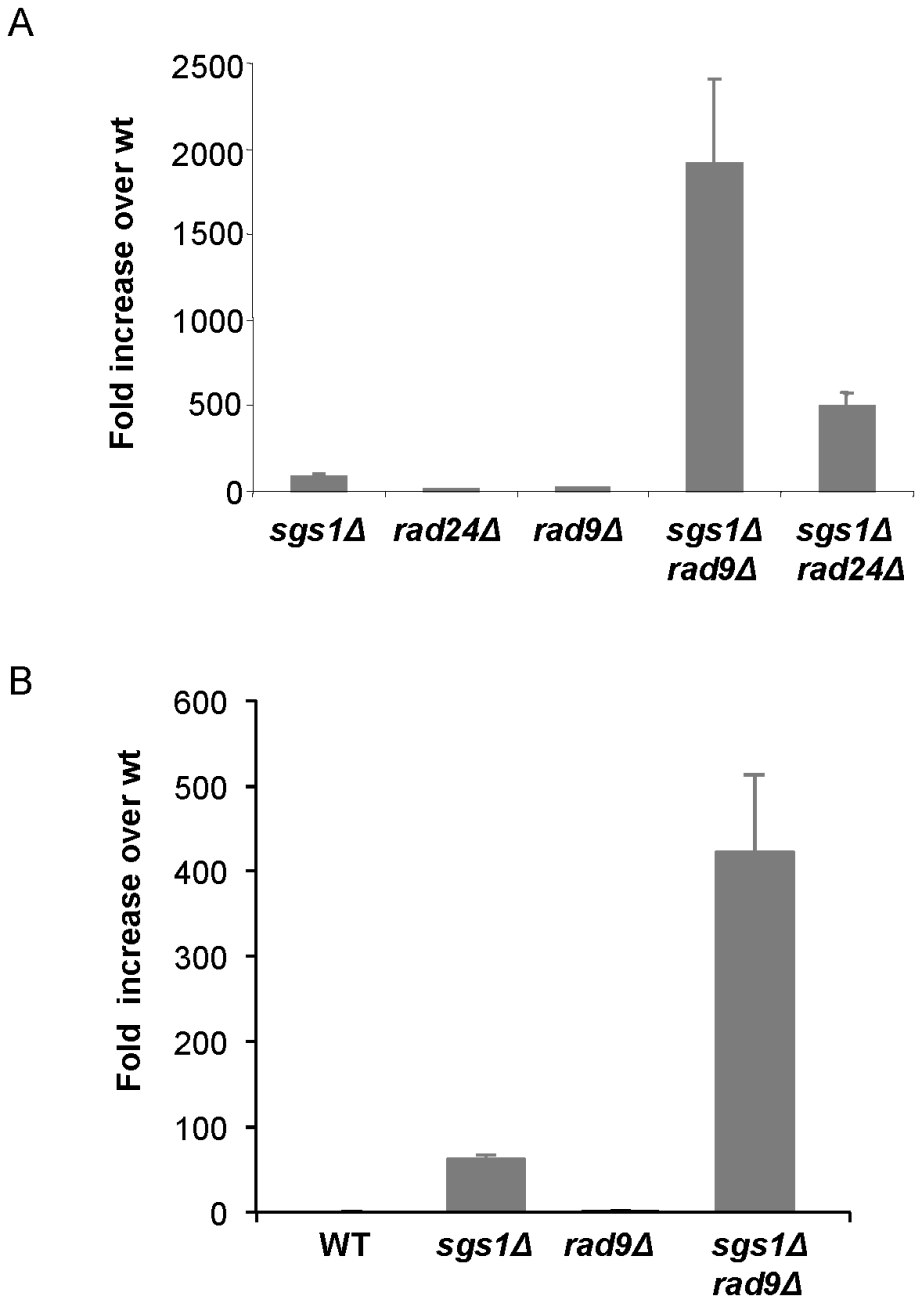
*sgs1Δ* induced genomic instability increases dramatically in *rad9Δ* cells as measured by Gross Chromosomal Rearrangements. (**A**) GCR were measured after exposure to 0.02% MMS, which results in 10% survival rate for the *sgs1Δrad9Δ* strain. GCR is shown as fold increase over wild type for the following strains: *sgs1Δ* (LBy-388), *rad24Δ* (LBy-406), *rad9Δ* (LBy-389), *sgs1Δrad9Δ* (LBy-400), *sgs1Δrad24Δ* (LBy-407). (**B**) Spontaneous GCR was measured for wild type (LBy-383), *sgs1Δ* (LBy-388), *rad9Δ* (LBy-389) and *sgs1Δrad9Δ* (LBy-400). GCR is shown as fold increase over wild type.

The recovery assays strongly suggest that Rad9 becomes critical, when Sgs1/Top3 is absent. To see if MMS treatment also leads to higher rates of genomic instability in *sgs1Δrad9Δ* cells as compared to *sgs1Δrad24Δ* cells, we performed a mutator assay that allows us to monitor the rate of gross chromosomal rearrangements (GCR). We performed GCR assays after exposure to 0.02% MMS, which results in a ∼10% survival rate for the *sgs1Δrad9Δ* strain in the S288C strain background (data not shown), the most sensitive of all mutants tested. Supporting the recovery data (Fig. 1), we observe synergism between *sgs1Δ* and *rad9Δ* for GCR rates (1900-fold over wild type, Fig. 2A), which is stronger than observed for the *sgs1Δrad24Δ* double mutant (490 fold over wild type, Fig. 2). Even in the absence of exogenous damage provoked by MMS, were we able to detect a synergistic effect when analyzing GCR rates for *sgs1Δ*, *rad9Δ* and *sgs1Δrad9Δ* cells (Fig. 2B). From this we conclude that genome integrity is more severely compromised in *sgs1Δrad9Δ* cells than in *sgs1Δrad24Δ* cells after treatment with MMS, and even in the absence of damage does *sgs1Δrad9Δ* cells display higher GCR rates compared to single mutants.

### Rad9 works parallel to Sgs1/Top3 in the MMS induced intra-S checkpoint response

The intra-S damage checkpoint is activated when replication forks encounter MMS-induced alkylation of DNA. This intra-S phase checkpoint is dependent on Rad53 and Mec1, and their primary task is to prevent irreversible replication fork collapse and inappropriate origin firing. Thus defects in the intra-S phase checkpoint response leads to loss of viability and faster S phase progression [44]. While it is well established that Rad9 works as a key activator of Rad53 in a G2 response to DNA double strand breaks and subtelomeric stretches of ssDNA [28], its role in the intra-S phase has been unclear. This is largely based on the fact that *rad9Δ* mutants only show partial defects in regulating S phase progression in response to MMS [45], [46]. Rad24 and members of the *RAD24* epistasis group (*RAD17*, *DDC1* and *MEC3*) exhibit a similar partial defect in S phase slowing [45], [47], [48]. Earlier analyses have shown that this partial defect in S phase slowing of *rad24Δ* cells is additive with an *SGS1* deletion [5], which is also reflected in the additive effect for recovery reported above. To investigate whether the synergism observed in the recovery assay and for GCR for the *sgs1Δrad9Δ* mutant can be explained by a severely compromised Rad53 checkpoint activation, we monitored Rad53 activation using the *in situ* autophosphorylation assay (ISA) [41].

**Figure 3:**
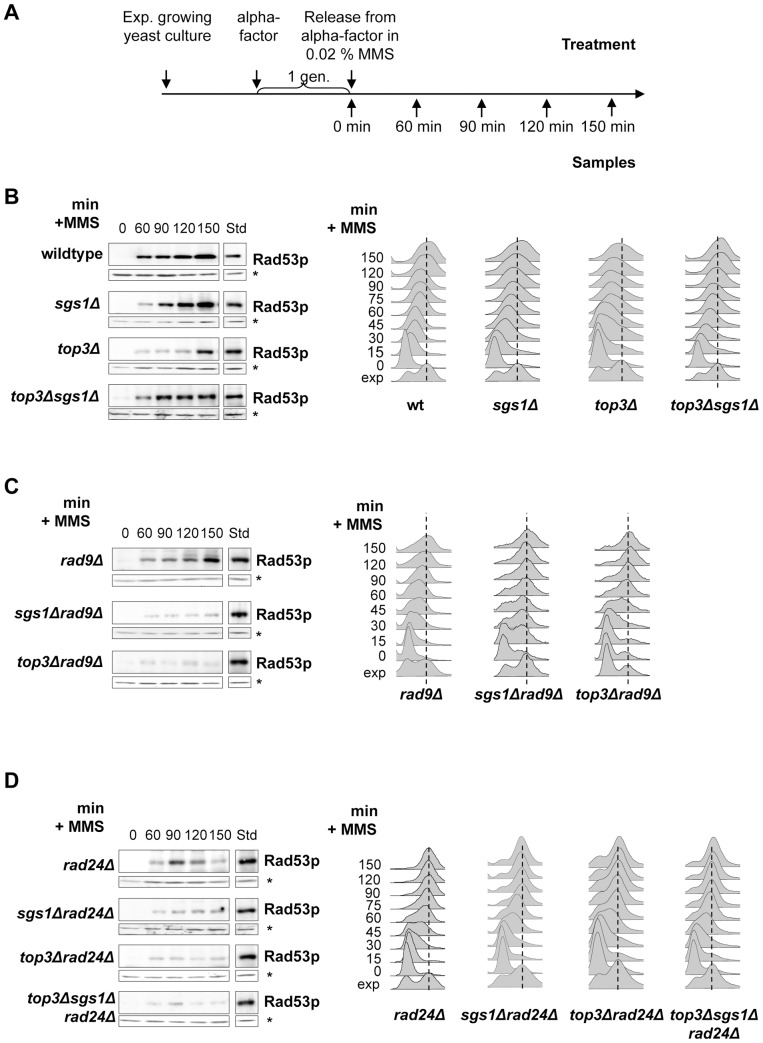
Rad9 works parallel to Sgs1/Top3 in the intra-S phase checkpoint response induced by MMS. (**A**) Experimental outline. (**B**) ISA analysis of Rad53 auto-phosphorylation measured for wild type (LBy-1), *sgs1Δ* (LBy-129), *top3Δ* (LBy-7) and *sgs1Δtop3Δ* (LBy-8). Cells were synchronised in G1, released into 0.02% MMS, and analysed at indicated times by ISA. For each strain the upper box shows the incorporation of γ^32^-ATP into Rad53, and the bottom panel a Western for RnaseH42 on the same blot (*). Time (min) after alpha-factor release is indicated above each panel. Std is 5 µl of a sample containing a fixed amount of activated Rad53 standard, which is used to normalise all gels after identical exposure times (see Materials and Methods). FACS samples were taken at 15 min intervals at the beginning of the experiment and 30 min intervals at the end of the experiment and shown on the right. (**C**) As in B but with the following strains: *rad9Δ* (LBy-316), *sgs1Δrad9Δ* (LBy-44) and *top3Δrad9Δ* (LBy-27). (**D**) As in B but with the following strains: *rad24Δ* (LBy-391), *sgs1Δrad24Δ* (LBy-36), *top3Δrad24Δ* (LBy-28) and *top3Δsgs1Δrad24Δ* (LBy-40).

Levels of Rad53 activation were compared for single-, double- and triple mutants and samples were taken for FACS analysis throughout the experiments to verify synchronization and S-phase progression. Cells were grown as outlined in Fig. 3A and extracts were prepared from samples taken at the indicated times. As evident from Fig. 3, the *rad9Δ* single mutant is much less compromised in Rad53 activation than is the *rad24Δ* single mutant, suggesting that the role of Rad24 is more central to proper checkpoint activation in otherwise wildtype cells. The strong delay or reduction observed for the *top3Δ* mutant is in agreement with an earlier report and likely reflects its reduced rates of S-phase entry and fork elongation as the checkpoint is restored in an *sgs1Δtop3Δ* mutant [22].

When a *rad9Δ* deletion is combined with either *sgs1Δ* or *top3Δ*, however, Rad53 activation on MMS is abolished (Fig. 3C). This resembles the result obtained with the *sgs1Δrad24Δ* double mutant, where the reduced Rad53 activation in *rad24Δ* cells is further compromised when combined with either *sgs1Δ* or *top3Δ* (Fig. 3D). This could suggest that when the Sgs1/Top3 alternative pathway is lost Rad9 has an essential co-activator role with Rad24. The simplest interpretation of these results is that Rad9 specifically amplifies the Rad53 response to MMS, which is not the case on HU [49]. Alternatively, a different type of damage may be generated on MMS in the absence of Sgs1/Top3, which renders checkpoint activation more dependent on Rad9. Interestingly, we note further that the involvement of Top3 differentiates this response to MMS from the response to forks stalled by HU, where we find no additive effect when combining a *top3Δ* with a *rad24Δ* [18].

From these checkpoint analyses we conclude that Sgs1 and Top3 act together on a secondary minor pathway that can activate Rad53 on MMS, parallel to that requiring Rad9, consistent with the observation that Sgs1 when phosphorylated by Mec1 directly binds Rad53 [20]. Thus, Rad9 and Top3/Sgs1 define two unequal but parallel pathways for checkpoint activation on MMS like what has been suggested for Sgs1 and Rad24 [5]. However, the highly synergistic effects on GCR or recovery seen between *sgs1Δ* and *rad9Δ* on MMS are not observed for *sgs1Δ rad24Δ* cells, thus compromised Rad53 activation is unlikely to be the explanation for this difference.

### Chk1 phosphorylation is equally compromised in *rad24Δ* and *rad9Δ* cells

**Figure 4:**
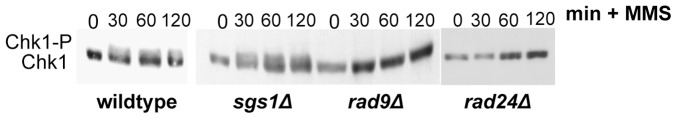
Chk1 activation is equally compromised in *rad24Δ* and *rad9Δ* cells. Chk1 upshift assay were performed to investigate checkpoint activation for the following strains: wild type (LBy-366), *sgs1Δ* (LBy-372), *rad9Δ* (LBy-374) and *rad24Δ* (LBy-390). Synchronized cultures of cells were released into S phase in the presence of 0.1% of MMS and aliquots were taken at the indicated times for analysis.

Rad9 is also required for *in vivo* Chk1 activation, although the role that Rad9 plays in this signal transduction from Mec1 to Chk1 is not as well understood as for the Mec1-Rad53 signal transduction pathway. Interaction between Rad9 and Chk1 has been shown by two-hybrid analysis [50], [51] and biochemical analysis have shown that it is the N-terminus of Rad9, which plays a role for Chk1 activation [25]. In *S. cerevisiae*, Chk1 seems to play a fairly minor role for the intra-S phase checkpoint activation, however, more recently it was shown that Chk1 can stabilize replication forks in the absence of Rad53 upon MMS induced damage [52]. To investigate if a more compromised Chk1 activation may explain the observed synergism between *sgs1Δ* and *rad9Δ*, we investigated Chk1 activation by upshift assay in *sgs1Δ*, *rad9Δ* and *rad24Δ* single mutants. Cells were synchronized in G1 by α-factor and released into S phase in the presence of 0.1% MMS. In this assay cells were treated with a higher concentration of MMS in order to activate Chk1. Levels of Chk1 phosphorylation were investigated in samples taken at different intervals after release. We find that absence of Sgs1 does not affect Chk1 phosphorylation, whereas cells lacking either Rad9 or Rad24 are equally compromised for Chk1 phosphorylation (Fig. 4). To exclude that that in absence of Sgs1 there are secondary lesions that activate Chk1 in a Rad9 dependent but Rad24 independent manner, we also analyzed Chk1 activation for the double mutants *sgs1Δrad9Δ* and *sgs1Δrad24Δ*. These analyses reveal that both strains are equally compromised in Chk1 activation (Figure S1). Taken together, our data on checkpoint activation suggest that the difference between *sgs1Δrad9Δ* and *sgs1Δrad24Δ* strains both for recovery from MMS induced damaged and suppression of GCR, is likely checkpoint-independent. This suggests that Rad9 may perform a checkpoint independent role in genome maintenance in the absence of Sgs1.

### A Rad53 checkpoint independent function of Rad9 is required for growth on MMS and suppression of GCR in cells lacking Sgs1

**Figure 5:**
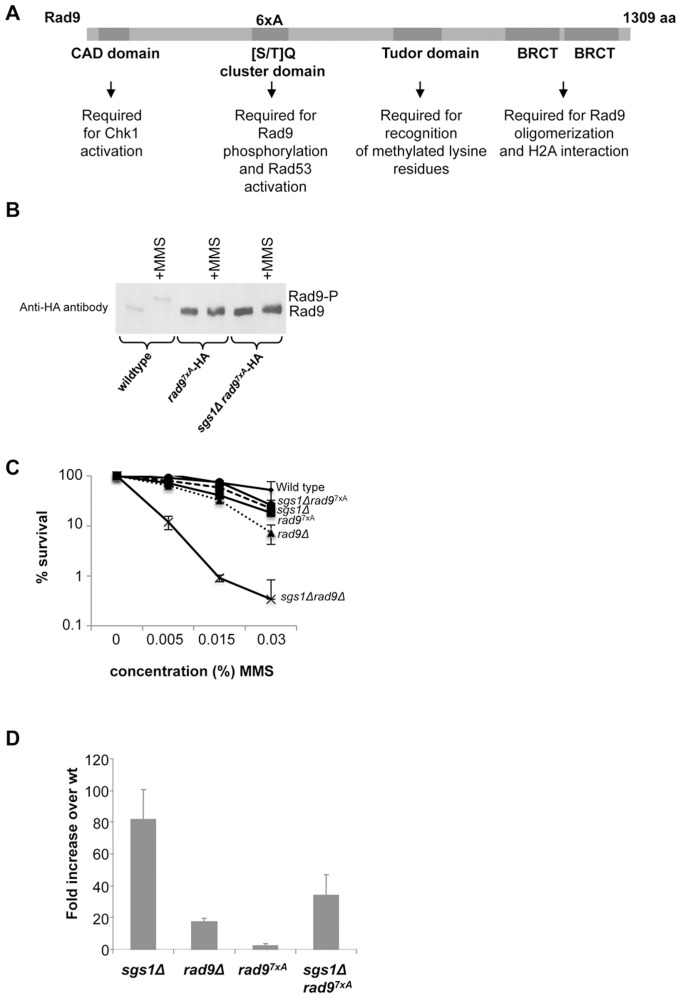
The Rad53 checkpoint function of Rad9 is not required for GCR suppression and growth in the presence of MMS in cells lacking Sgs1. (**A**) Illustration of the domain structure of Rad9. (**B**) Immunoprecipitations were conducted with extracts from the contructed *rad9 ^7xA^-*HA strain (LBy-471) and the *sgs1Δrad9 ^7xA^*-HA strain (LBy-472) to verify the 7xA mutations. Immunoprecipitations were performed with anti-HA antibody in the presence and absence of MMS. (**C**) Survival was monitored as described in Materials and Methods after 70 min exposure to different concentrations of MMS for the indicated strains: wild type (LBy-1), *sgs1Δ* (LBy-129), *rad9Δ* (LBy-316), *sgs1Δrad9Δ* (LBy-44), *rad9 ^7xA^* (LBy-471), *sgs1Δ rad9 ^7xA^* (LBy-472). (**D**) GCR were measured after exposure to 0.02% MMS and is shown as fold increase over wild type for the isogenic strains: *sgs1Δ* (LBy-388), *rad24Δ* (LBy-406), *rad9Δ* (LBy-389), *rad9 ^7xA^* (LBy-473), *sgs1Δ rad9 ^7xA^* (LBy-474).

To further investigate a possible checkpoint independent role of Rad9 for genome maintenance, we took advantage of a Rad9 mutant defective for Rad53 activation, *rad9* ^7xA^ [53]. This mutant carries alanine substitutions at each [S/T] Q within the [S/T] Q cluster domain (SCD) and furthermore has an alanine substitution of T603 (Fig. 5A). The *rad9* ^7xA^ is unable to undergo phosphorylation and interact with Rad53 [53].

We generated a *rad9* ^7xA^-HA and an *sgs1Δrad9* ^7xA^-HA strain. The strains were confirmed by immunoprecipitation, which verifies that the mutant form of Rad9 does not lead to an upshift upon MMS induced damage, whereas wild-type Rad9 does (Fig. 5B). Furthermore, Rad53 kinase assay confirms that Rad53 autophosphorylation is similar in the *rad9Δ* and *rad9* ^7xA^-HA strains (Figure S2). Next we investigated survival using the MMS recovery assay also used for Fig. 1. From this it becomes evident that the double mutant *sgs1Δrad9* ^7xA^-HA displays approximately the same sensitivity to MMS as an *sgs1Δ* mutant and is much less sensitive than the *sgs1Δrad9Δ* mutant, thus the checkpoint function of Rad9 is not required for growth in the presence of MMS when Sgs1 is absent (Fig. 5C). This reinforces our conclusion that it is not the checkpoint function of Rad9, which is crucial in the absence of Sgs1. To investigate whether this apparently checkpoint independent function of Rad9 also accounts for the synergism observed for GCR in the *sgs1Δrad9Δ* double mutant, we measured GCR for the *sgs1Δrad9^7xA^* double mutant and compared this with the single mutants. Interestingly, the Rad53 checkpoint function of Rad9 is not required for GCR suppression in the absence of Sgs1, as *sgs1Δrad9^7xA^* shows lower levels of GCR than a *sgs1Δ* mutant, substantiating that a checkpoint independent role of Rad9 becomes essential for genome maintenance in the absence of Sgs1. So far we can only speculate why a *rad9* ^7xA^ partly suppresses the GCR of an *sgs1Δ* mutant. In the *sgs1Δ* mutant Rad9 is expected to bind Rad53 upon MMS treatment, whereas this will not be the case in the *sgs1Δrad9^7xA^* double mutant. Whether this “frees” Rad53 for other interactions, which are more beneficial for GCR suppression in the absence of Sgs1 can be one explanation. However, this will need further investigations.

It is furthermore interesting to note, that the *rad9^7xA^* mutant has less severe phenotypes compared to *rad9Δ* both concerning recovery after MMS and MMS induced GCR, which could indicate that although Rad9 is identified as a checkpoint protein, its Rad53 activating role is of minor importance for survival and genome stability upon MMS induced damage compared to other potential roles of Rad9.

## Discussion

In this study we have investigated a possible genetic interaction between the *SGS1* and *RAD9*. Our study reveals several novel insights into how cells respond to MMS induced damage. First, we demonstrate a strong synergistic functionality between Sgs1/Top3 and Rad9 for recovery from MMS damage and suppression of GCR, which is not seen between Sgs1/Top3 and Rad24. These observations suggest that cells rely on a Rad9-dependent pathway in the absence of Sgs1 that is at least partially independent of Rad24, and we confirm that this is a Rad53 checkpoint independent function of Rad9. Second, our dissection of the MMS checkpoint response discloses parallel, but unequal pathways for Rad53 activation and furthermore highlights significant differences between MMS and HU induced checkpoint responses with relation to the requirement of Sgs1/Top3. Thus, our checkpoint analyses, which are based on Rad53 activation, confirm that Sgs1/Top3 defines a minor but parallel pathway to that of Rad9 during an MMS response. It has previously been suggested based on S phase progression analysis, that Sgs1 defines a pathway parallel to that of Rad24 [5]. Since *rad9Δ* is less compromised in checkpoint activation than a *rad24Δ*, our data clearly demonstrates that Sgs1/Top3 defines a second checkpoint pathway as the double mutants *sgs1Δrad9Δ* and *top3Δrad9Δ* are significantly more compromised in Rad53 activation than either single mutants. Interestingly, earlier studies have shown that Top3 is not engaged in a checkpoint pathway in response to HU, there Sgs1 works independently of Top3, however in the absence of either Rad9 or Rad24 checkpoint activation depends on both Sgs1 and Top3.

Treatment with MMS induces methylated DNA lesions, which are predominantly repaired by the base excision repair (BER) pathway and DNA alkyltransferases [54]. For a long time it was generally assumed that MMS damage led to DSB formation. This assumption was based on several facts; homologous recombination (HR) deficient cells are sensitive to MMS and DNA fragmentation was observed after MMS treatment by pulsed-field gel electrophoresis (PFGE). However, more recent it was demonstrated that DNA fragmentation following MMS treatment is an artefact arising during sample preparation [55], thus the requirement of HR for survival after MMS treatment may more likely reflect a function for HR in the repair of MMS-induced stalled forks, although this still needs to be proven experimentally. If MMS damage is introduced during S phase this can indeed have a huge impact on replication forks, as these will stall when encountering the damage. The stalled replication fork may regress to form a chicken foot structure and remain stable until the damage has been removed by BER or the lesion may be tolerated, which would rely on either template switching or translesion synthesis. If forks regress, it is fairly easy to imagine a function of Sgs1/Top3. Sgs1/Top3 may either be involved in reversal of regressed forks, fork stability or if the regressed fork undergo homologous recombination to restore the replication fork, Sgs1/Top3 would be needed for the dissolution of the formed dHJ.

How may the genetic interaction between Sgs1 and Rad9 be explained? In a genetic screen for interactions with *rad9Δ* that confer sensitivity to MMS several proteins engaged in post-replication repair both in the error-free template switching pathway and the error-prone translesion synthesis pathway were identified. Furthermore, also enzymes engaged in HR resolution were identified such as Mus81 and Sgs1 [35]. These genetic interactions could indicate that replication forks encounter more MMS induced damage in the absence of Rad9, and thereby require pathways to deal with replication fork stalling or bypass of the damage. To account for this explanation Rad9 should have a direct function in the repair of alkylated damage or as have previously been suggested it should actively promote the use of BER. Rad9 has been implicated in nucleotide excision repair of UV-damaged DNA [33], [34], however so far there are no reports on a function of Rad9 in BER.

In 1995 it was reported that Rad9 had a different role than Rad17, Rad24 and Mec3. Where the latter checkpoint proteins activated an exonuclease upon damage, which would degrade the DNA, Rad9 was found to inhibit DNA degradation [56]. Following damage near telomeres, Rad9 was shown to play both a checkpoint dependent and independent role for suppression of ssDNA formation. Based on genetics a model was suggested, where Rad9 in a pathway with Mec1 and Rad53 inhibits the action of Exo1 and in a pathway independent of Mec1 and Rad53 inhibits another exonuclease [57]. These data are suggestive of a “protective” role of Rad9. Further investigations have revealed that inhibition of ssDNA at uncapped telomers as well as at DSBs by Rad9 invovle interaction between the Tudor domain of Rad9 and methylated histone H3-K79 [58] Also the tumor suppressor p53-binding protein 1 (53BP1) interacts with histones via its Tudor domain. This interaction requires the dimethylated form of histine H4 K20 [59]. More recent it was furthermore shown that 53BP1 also controls ssDNA formation at dysfunctional telomeres as well as at DSBs by inhibiting resection, and this process is dependent on Rif1 [60], [61], [62]. In further support of a Rad53 checkpoint independent function of Rad9, a post checkpoint activation role of Rad9 in promoting efficient repair of DNA DSBs by homologous recombination induced by irradiation has been reported [36]. In this study it was shown that Rad9 foci colocalise with a subset of Rad52 foci and that the hypophosphorylated form of Rad9 associates with damaged chromatin.

Rad9 shares several features with the tumour suppressor protein BRCA1, most notably the presence of BRCT domains [53], [63]. BRCT domains have been implicated in the recognition of H2AX (or phospho-H2A in yeast), which is found both at sites of double strand breaks, as well as at stalled forks [19], [64], and which has been suggested to be necessary for efficient repair [65]. Moreover, BLM helicase has been shown to associate with *γ*H2AX in response to Top1-mediated strand breaks [66]. Thus it is possible that BRCT domain-containing proteins play a role in repair as well as checkpoint processing, perhaps by recruiting proteins to sites of damage. For instance, BRCA1 has been implicated in acting as a scaffold, as well as processing abnormal DNA structures [23], [67]. Such a role is conceivable as well for Rad9, which may act in parallel to Sgs1-mediated recombinational repair at MMS-induced damage. To this end, we have found an interaction between Rad51 and Rad9 *in vivo* (data not shown).

The first step in S-phase checkpoint activation involves replication fork stalling, either due to the depletion of dNTPs by HU or due to encountering of alkylated DNA induced by MMS. However, although the initial step is the same, the downstream events are likely to differ depending on the destiny of the replication fork. This current study clearly shows that there are significant molecular differences between a response provoked by MMS and one provoked by HU. This is illustrated by the different requirements for Rad9 depending on the type of fork stalling. On HU, Rad53 activation can be achieved in the absence of Rad9, however upon loss of either Tof1 or Mrc1, Rad53 activation becomes Rad9-dependent [49], [68]. Under MMS conditions on the other hand, Rad53 activation is Rad9-dependent (this study and [46]). This suggests that upon stalling by HU, cells engage a “stabilization pathway” that relies on Mrc1 and Tof1, and as we have shown in the past, Sgs1 and Mec1 kinase [17], [18]. If stabilization cannot be achieved, the stalled replication fork is converted to another form of damage or structure, and checkpoint activation now reflects a Rad9-dependent pathway. We propose that in the presence of MMS-induced damage, replication forks adopt a form different than during HU treatment and this form relies on the Rad9 pathway for checkpoint activation. Interestingly, the requirement for Top3 further differentiates between HU and MMS induced checkpoint activation. In an earlier report, we found that on HU, the cells engage a Sgs1-dependent checkpoint activation. Although a reduced checkpoint signal is only evident in *rad24Δ* cells, we could show that the Sgs1-dependent activation of Rad53 did not require neither Top3 nor Rad51, and an enzymatic active Sgs1 was also not required [18]. Indeed this is consistent with data showing that Sgs1 binds the Rad53 kinase FHA1 domain directly when an acidic domain upstream of the Sgs1 helicase domain is phosphorylated by Mec1 [20]. We further suggested that the Rad24 and Sgs1 pathways are not simply redundant, but act preferentially at different types of S-phase lesions [18].

Our MMS checkpoint analysis enables us to further extend this model. Checkpoint activation here relies almost entirely on Rad24, and not on Sgs1. However, we can detect a secondary pathway, which becomes especially clear when comparing *rad9Δ* and *sgs1Δ* single mutants with the *sgs1Δrad9Δ* double mutant, that requires both Sgs1 and in this case, its interacting partner Top3. This distinguishes it from the role of Sgs1 in the presence of fork arrest induced by high HU concentrations. These data suggest that Sgs1 is engaged in distinct pathways depending on the type of damage, or that in its absence, different damaging agents have different outcomes.

This study identifies a Rad53 checkpoint independent role of Rad9 crucial for genome maintenance in the absence of the RecQ helicase Sgs1, although we also show that Rad9 and Sgs1/Top3 work in parallel but unequal pathways for Rad53 activation during MMS. Our data and previous analysis on Rad9 strongly points to this enzyme maintaining multiple functions for genome integrity, which is not only embedded in its checkpoint role. A precise dissection of these checkpoint independent roles of Rad9 will be an intriguing challenge for future studies.

## Supporting Information

Figure S1 **Chk1 upshift assay were performed to investigate checkpoint activation for the following strains: wild type (LBy-366),** ***sgs1Δ*** **(LBy-372),** ***rad9Δ*** **(LBy-374),** ***rad24Δ*** **(LBy-390),** ***sgs1Δrad9Δ*** **(LBy-376) and** ***sgs1Δ rad24Δ*** **(LBy-1085).** Experiments were performed as described in Material and Methods and in the same way as the experiment shown in Figure 4, except that only one time point is loaded on these gels in order to have all single mutants on the same gel. Wild type (60 min) shown on the lower gel is the same as wild type (60 min) on the upper gel. This has been included as a reference for upshift, when investigating the double deletion mutants.
(TIFF)

Figure S2 **Rad53 kinase assay were performed to compare Rad53 activation in a** ***rad9Δ*** **(LBy-316) and a** ***rad9^7xA^*** **(LBy-471) strain.** The experiment was performed as described in Material and Methods and in the same way as the experiments shown in Figure 3 except in this experiment Mcm2 was used as loading ctrl. Std is 5 µl of a sample containing a fixed amount of activated Rad53 standard.
(TIFF)
