# Supplementary figures and images for: A Rad53 Independent Function of Rad9 Becomes Crucial for Genome Maintenance in the Absence of the RecQ Helicase Sgs1
Source: PLoS One. 2013 Nov 20;8(11):e81015. doi: 10.1371/journal.pone.0081015 (PMC3835667; doi:10.1371/journal.pone.0081015)

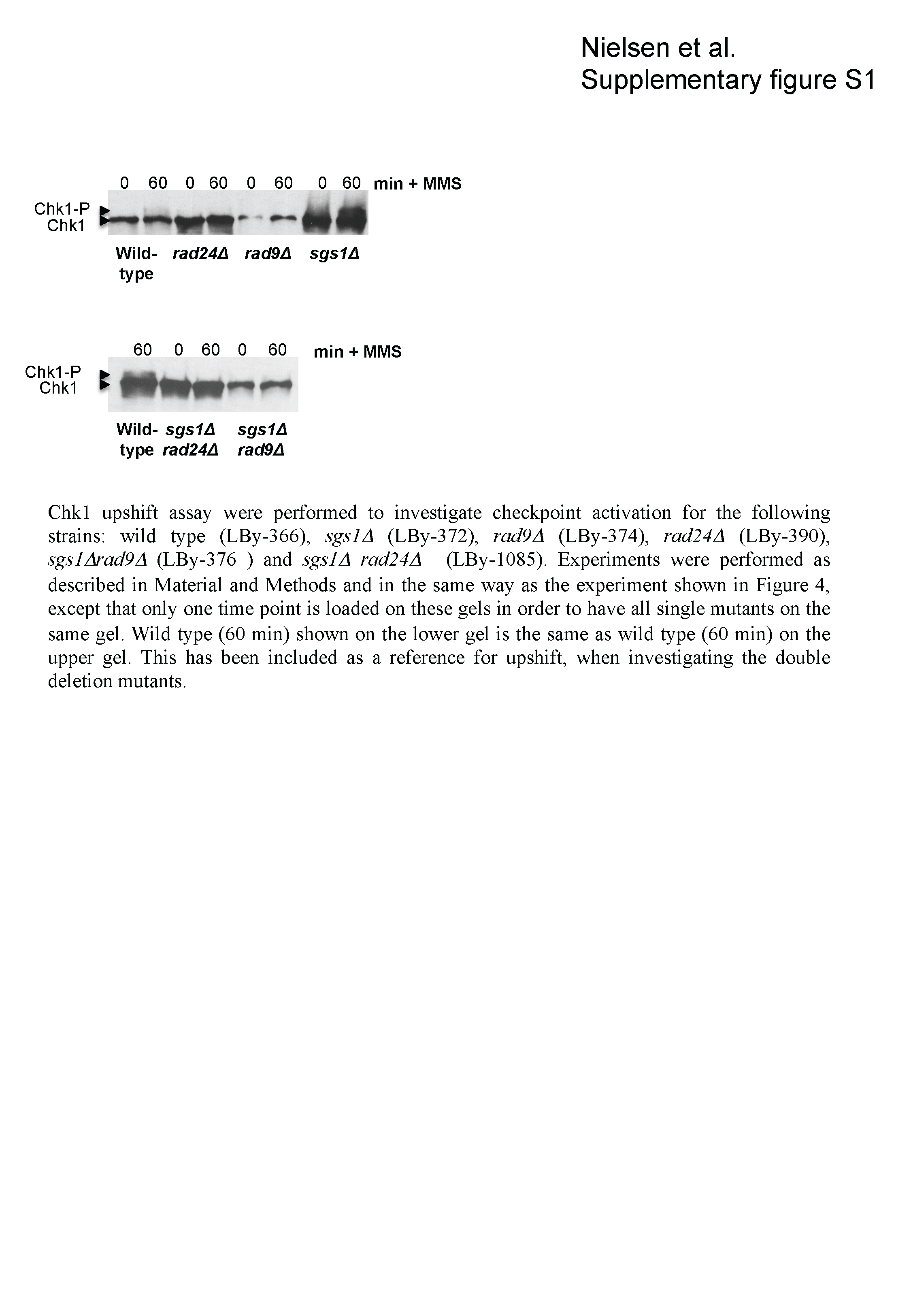

Supplement: Figure S1 — Chk1 upshift assay were performed to investigate checkpoint activation for the following strains: wild type (LBy-366), sgs1Δ (LBy-372), rad9Δ (LBy-374), rad24Δ (LBy-390), sgs1Δrad9Δ (LBy-376) and sgs1Δ rad24Δ (LBy-1085). Experiments were performed as described in Material and Methods and in the same way as the experiment shown in Figure 4, except that only one time point is loaded on these gels in order to have all single mutants on the same gel. Wild type (60 min) shown on the lower gel is the same as wild type (60 min) on the upper gel. This has been included as a reference for upshift, when investigating the double deletion mutants. (TIFF) [file pone.0081015.s001.tiff]
